# Transcatheter aortic valve replacement failure: a case report of the bicuspid aortic valve type 0 with a single coronary artery

**DOI:** 10.1093/jscr/rjac099

**Published:** 2022-03-21

**Authors:** Ryo Suzuki, Masato Suzuki, Ryo Takayanagi, Shunsuke Ohori, Toshiro Ito

**Affiliations:** Department of Cardiovascular Surgery, Ohno Memorial Hospital, Hokkaido, 2-1-16-1 Miyanosawa, Nishi-ku, Sapporo 063-0052, Japan; Department of Cardiovascular Surgery, Ohno Memorial Hospital, Hokkaido, 2-1-16-1 Miyanosawa, Nishi-ku, Sapporo 063-0052, Japan; Department of Cardiovascular Surgery, Ohno Memorial Hospital, Hokkaido, 2-1-16-1 Miyanosawa, Nishi-ku, Sapporo 063-0052, Japan; Department of Cardiovascular Surgery, Ohno Memorial Hospital, Hokkaido, 2-1-16-1 Miyanosawa, Nishi-ku, Sapporo 063-0052, Japan; Department of Cardiovascular Surgery, Ohno Memorial Hospital, Hokkaido, 2-1-16-1 Miyanosawa, Nishi-ku, Sapporo 063-0052, Japan

## Abstract

Transcatheter aortic valve replacement (TAVR) is the treatment of choice for aortic stenosis. However, its safety and efficacy in patients with the bicuspid aortic valve (BAV) remain controversial. Especially, whether the BAV phenotype affects outcomes following TAVR remains debated. Despite the higher ellipticity index and more calcifications of the aortic annulus in type 1 BAV, a high residual gradient was observed in type 0 anatomy. Moreover, severe calcification of the cusps rather than aortic annulus in type 0 is predisposed to asymmetrical under-expansion of the prosthesis at the edge of the native aortic cusp. We report the rare case of a patient with BAV stenosis type 0 and single coronary artery receiving TAVR, subsequently requiring surgical aortic valve replacement. The extensive non-coronary cusp calcification caused under-expansion of the prosthesis and was protruded into the left ventricular outflow tract, leading to an obstruction.

## INTRODUCTION

Recent evidence proposes that transcatheter aortic valve replacement (TAVR) is a promising alternative to surgical aortic valve replacement (AVR) for not only high risk but low-risk patients [1, 2]. Alternatively, bicuspid aortic valve (BAV) has an asymmetrical valve orifice and are often associated with heavy regional protruding calcifications, abnormal coronaries take-off and aortopathy. All these anatomical features increase the risk of complication such as coronary obstruction, paravalvular leaks (PVL), annular rupture [5]. For these reasons, BAV was initially considered a relative contraindication to TAVR. However, ~20% of elderly patients with severe aortic stenosis have BAV. In these patients, TAVR is considered a treatment option in cases of intermediate-to-high surgical risk [3]. Moreover, recent study showed that comparing with tricuspid aortic stenosis, TAVR in bicuspid aortic stenosis was associated with a similar 2-year mortality and morbidity with new generation device [6]. Nevertheless, whether BAV phenotype affects outcomes following TAVR remains unclear [3].

## CASE REPORT

**Figure 1:**
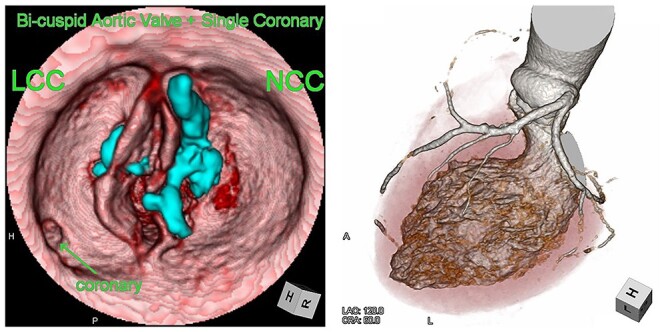
Computed tomography images of the bicuspid aortic valve type 0 and single coronary artery. Heavy calcification on NCC is indicated in blue.

**Figure 2:**
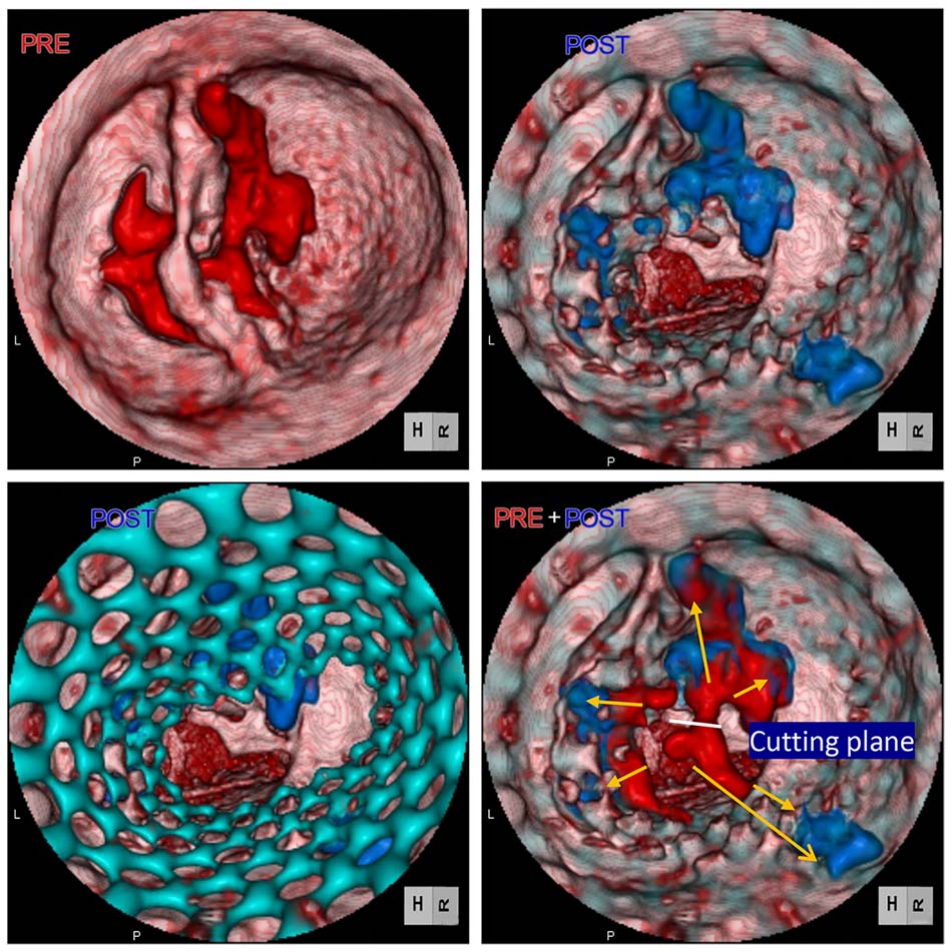
Computed tomography images of calcification before (red) and after (blue) TAVR. Distorted THV (green) at the annulus level was noted. Images were merged to detect the shifting calcification.

We report the case of an 82-year-old female with severe BAV stenosis type 0 and single coronary artery (Fig. 1). Given the risk profile and her preference, the decision was made for TAVR rather than surgical AVR. In the operating room (OR), we barely advanced the hard wire across the aortic valve due to the extensive calcification on the non-coronary cusp (NCC). Finally, a transcatheter heart valve (THV) was deployed under fibrillatory arrest, however the expansion of THV was suboptimal especially around the lower portion of THV close to the aortic annulus probably due to the hard extensive NCC calcification (Fig. 2). The balloon valvuloplasty was performed and she was taken back to the intensive care unit. Given the renal failure and patient’s age, further intervention was not recommended at this point.

**Figure 3:**
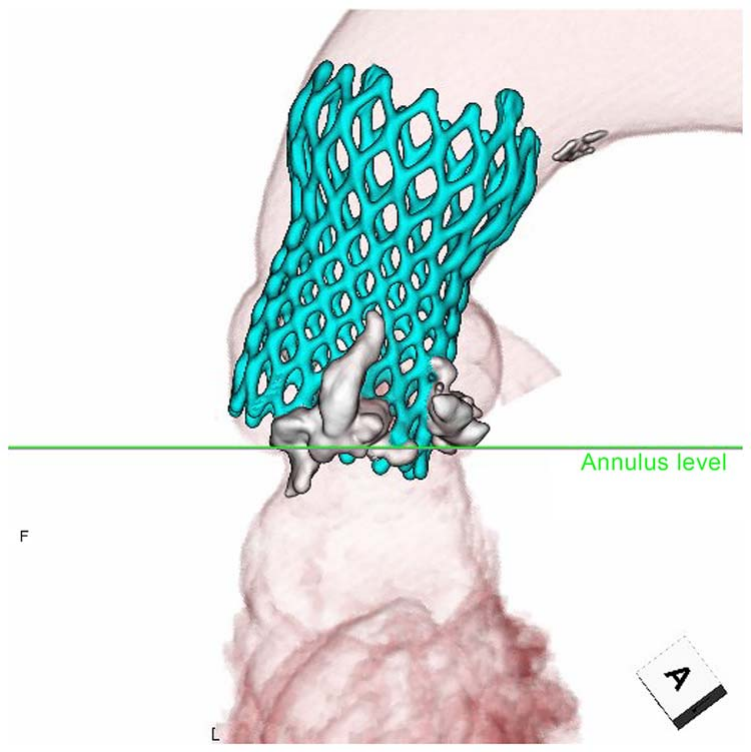
Migration of THV. Tilted prosthesis at the NCC annulus.

Postoperative echocardiography revealed a high transprosthetic gradient as well as moderate PVL. Alternatively, multidetector computed tomography (MDCT) revealed THV migration, tilted and elevated around the NCC annulus (Fig. 3). MDCT also identified the calcification location before and after TAVR (Fig. 2). Those two different calcification locations were merged into one image to clarify the shifting calcification. The calcification was shifted considerably around the NCC area of the annulus, consistent with the migration area of THV.

**Figure 4:**
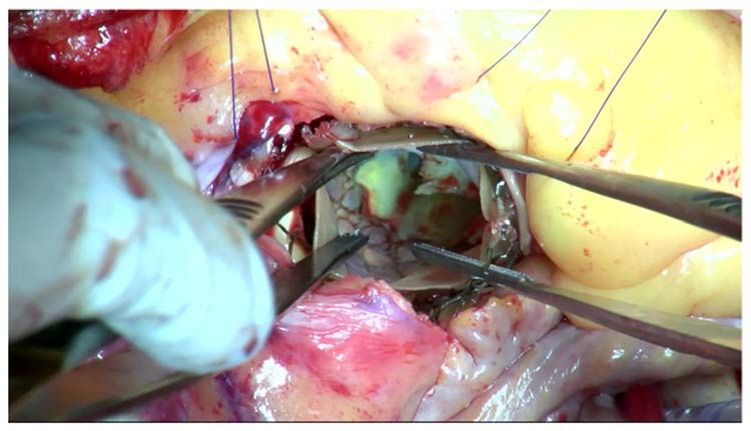
The protruding calcification into LVOT was identified in the OR.

**Figure 5:**
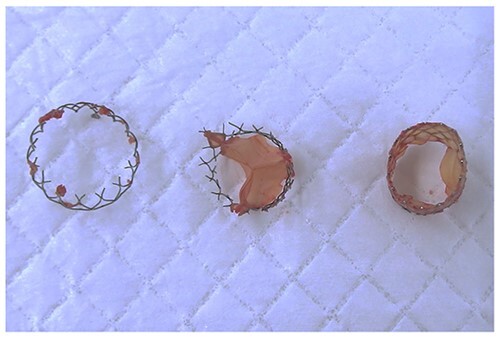
Extracted THV in pieces.

Moreover, the NCC calcification was shifted and protruded into the left ventricular outflow tract (LVOT), leading to an obstruction, as observed in the OR during the redo surgical AVR conducted due to the high transprosthetic gradient and PVL (Fig. 4). Subsequently, THV was extracted piece by piece, starting from the bare metal portion at the top followed by the frame and leaflet (Fig. 5). Then, decalcification was performed particularly around the NCC annulus and a bioprosthesis was implanted.

## DISCUSSION

There have been conflicting reports on the impact of different anatomical BAV variants on clinical outcomes. Mylotte *et al.* [7] found type 1 BAV to be associated with a higher rate of moderate–severe aortic regurgitation (regurgitation was defined as the sum of transvalvular and paravalvular regurgitation) compared to other BAV anatomies after TAVR, whereas Yousef *et al*. [8] showed that type 1 BAV anatomy (particularly L–R subtype), was associated with significantly lower 30-day and 1-year mortality and a higher device success, and lower rate of moderate–severe aortic regurgitation after TAVR. Studies have showed that type 0 BAV was strongly associated with a higher residual transprosthetic pressure gradient than other BAVs after TAVR. As observed, type 0 BAV had no raphe with extensive calcification on the leaflet than the aortic annulus. Therefore, THVs were less prone to expand, resulting in higher gradients secondary to valve asymmetrical expansions [3, 4]. In general, when deploying the valve, THVs expand and break the aortic leaflet to weak points, such as commissures/raphes. However, as for type 0 BAV, there was no raphe and leaflets were well-calcified to the point where they did not easily break with the balloon valvuloplasty, resulting in a higher gradient. For these reasons, we planned to place the THV at the supra-annular position for a better effective orifice area. Although the extensive NCC calcification was shifted and separated away during the procedure, one half of fragment was protruding into the LVOT, causing the obstruction and high transprosthetic pressure gradient. Responsible causes for failure were under-expansion of THV due to BAVs, supra-annular THV implantation (possibly causing the migration) and the extensive NCC calcification which should have been shifted toward the sinus of Valsalva rather than the LVOT. The evidence showed the postprocedural device success was lower in type 0 vs. type 1 BAV (72% vs 86.7%; *P* = 0.07) [3] although type 1 BAV had a higher ellipticity index and more calcifications of the aortic annuls. Despite these differences in device success and high residual pressure gradient, 1-year mortality was comparable among type 0 and 1 BAV in this study [3]. Taking these features together, careful attention should be paid to BAV phenotype when considering TAVR. Hence, straightforward surgical AVR is still reasonable option for complicated BAVs.
